# Dependence of credit spread and macro-conditions based on an alterable structure model

**DOI:** 10.1371/journal.pone.0196792

**Published:** 2018-05-03

**Authors:** Yun Xie, Yixiang Tian, Zhuang Xiao, Xiangyun Zhou

**Affiliations:** 1 School of Management and Economics of UESTC, Chengdu, China; 2 School of Mathematics and Statistics, Guizhou University, Guiyang, China; University of Toronto, Rotman School, CANADA

## Abstract

The fat-tail financial data and cyclical financial market makes it difficult for the fixed structure model based on Gaussian distribution to characterize the dynamics of corporate bonds spreads. Using a flexible structure model based on generalized error distribution, this paper focuses on the impact of macro-level factors on the spreads of corporate bonds in China. It is found that in China's corporate bonds market, macroeconomic conditions have obvious structural transformational effects on bonds spreads, and their structural features remain stable with the downgrade of bonds ratings. The impact of macroeconomic conditions on spreads is significant for different structures, and the differences between the structures increase as ratings decline. For different structures, the persistent characteristics of bonds spreads are obviously stronger than those of recursive ones, which suggest an obvious speculation in bonds market. It is also found that the structure switching of bonds with different ratings is not synchronous, which indicates the shift of investment between different grades of bonds.

## Introduction

Bonds market is an important component of China’s financial market. But due to historical reasons of the economic system, China’s bonds market has been developing slowly with respect to China’s stock market. The bonds market is small and has low degree of marketization. By the end of 2006, the balance of the China’s bonds market was CNY 5745.5 billion, 27.44% of GDP, far below the level of 163.11% in developed countries. In particular, the corporate bonds balance is only CNY 283.1 billion, accounting for 5% of the whole bonds market share, and only 1.35% of GDP, or even lower than the level of 30% in the Asian countries with financial crisis. After 2007, the Chinese government decided to vigorously develop the corporate bonds market, implemented the approval system of bonds issuance, and gradually formulated a series of regulations, which indicated a new stage for the issuance of corporate bonds. Since then, corporate bonds have begun to develop rapidly. By the end of 2016, the balance of China's bonds market reached CNY 63.7 trillion, only lower than the Unites States and Japan. The credit balance of corporates of China was CNY 16.5 trillion, which was only lower than that of the Unites States. So it is of great practical significance to study the China’s bonds market at this time.

Bonds market is the most important channel for enterprises financing, and spreads is the embodiment of bonds risk. Spreads directly affects the refinancing opportunities and the financial structures of enterprises and has a far-reaching impact on the decision-making, profitability and growth of enterprises. The bonds spreads are theoretically defined as the difference between the bonds yield and the risk-free rate, but in practice, the risk-free rate is often substituted by the Treasury bonds yield of the same-term structure. As the risk compensation for defaultable securities, the determinants of spreads are various and have always been the focus of relevant studies.

In essence, profitability determines the probability of an enterprise defaulting on its outstanding debt, which is also the determining factor of bonds spread, so the early bonds structure models were developed [1–3]. But since then, it has been increasingly found that corporate bonds spreads are largely influenced by factors such as macro economy, financial market and monetary policies, etc. In fact, the value of an enterprise is the key factor of whether the enterprise default impacts the bonds or not, and the value depends on the future cash flow and the corresponding discount rate, both of which are closely related to the economic environment of the enterprise. Thus, dynamic capital structure model was used to reveal the impact of macroeconomic condition on corporate bonds spreads [4–6]. On the other hand, analysis was performed to investigate the roles of macro economy in the risk of bonds default [7, 8], or examine the effect of macroeconomic conditions on corporate bonds default decisions from the perspective of default costs [9]. Considering the relationship between stock market and bonds market, many attempts have been made to find an explanation for the changes in bonds spreads from the stock market [10–12]. Meanwhile, factors such as money market and investor sentiment have also received considerable attentions [13, 14]. Although bonds spreads have been studied from various angles, it is usually assumed that the model has a stable structure over the sample period, namely that the loads of explanatory variables are fixed. However, the cyclical nature of the economy has been extensively confirmed. Both bonds market and enterprises are affected by the economic cycle in different ways. Furthermore, changes in monetary, fiscal, legal and other relevant policies or regulations can also affect the operation of an enterprise and the bonds market, which in turn will cause the fluctuation of bonds spreads. The occasional default events also cause a change in the risk appetite among market investors, resulting in a surge in bonds spreads. Therefore, there is a disconnection between the changes of variables that theoretically affect credit spreads and credit spreads fluctuations. Hence, studying the influencing factors of credit spreads using an invariant model structure is not in accordance with the actual condition and hinders the understanding for the law of spreads fluctuations.

The switching of the model structure is usually unobservable and may endogenously depend on one or more variables, which is usually assumed to have Markov properties. Hamilton and Susmel applied the Markov switching model to stock analysis for the first time [15]. Timmermann further explored the econometric properties of the model and provided methodological support for studying the structural transformation of macro economy and financial markets [16]. Then, Giesecke et al. found the periodic default on corporate bonds through a study of 150 years' history of U.S. bonds [17]. However, they only considered the structural transformation of the model intercept. Ghysels et al. used structural switching model to identify the obvious structural changes between risk and yield [18]. Based on enterprise level considerations, Chun et al. investigated the cyclical impact of default rate and liquidity on bonds spreads [19], while Chun et al. analyzed the credit supply, monetary policy and economic cycle and other factors’ local impact on the spreads by considering the structure change, [20], but ignored the existence of interaction among various factors that might affect the structural switching, and then drew a different conclusion.

This paper is similar to [19, 20] in terms of method or content, but due to the large difference in the corporate bonds market between China and the United States, this paper is distinctly different from them. China's economy has exceeded the United States from the scale (PPP), but the development of market economy and financial market are at an early stage, and the corporate bonds market was even established about ten years ago. In spite of its rapid development, the scale of bonds market is relatively small compared with other financial markets such as stocks, so it is vulnerable to various factors such as regime, money supply and other financial market fluctuations. Although China has implemented the bonds issuance approval system since 2007, the high threshold of bonds issuance has made the bonds rating very high for a long time, for example, the newly-issued bonds in 2016 generally had a high rating (AAA31.6%, AA+26.2%, AA39.4%, AA-2.6%). Therefore, the actual default rate of the bonds is very low, and the price of the bonds is more influenced by macro factors than corporate characteristics. Chun et al. focused on the cyclical impact of the United States’ macro-level factors on corporate bonds [20], but due to the immature market in China, the situation in China is much more complicated. China's corporate bonds spreads are not only influenced by money supply, institutional change, stock market, also by its own market, so a correct understanding of the factors affecting the bonds spreads is helpful for China to improve and manage the bonds market, and contributes to investors’ clear identification of bonds investment risk. Therefore, in view of the characteristics of China's bonds market, this paper analyzes the influence of multiple macro-level factors on corporate bonds spreads in the context of structural transformation. The differences between this paper and [20] are: 1, due to the data characteristics, the structural transformation model of this paper is based on a generalized error distribution (GED); 2, this paper comprehensively considers the structural transformation characteristics of multiple macro-level factors, while [20] focused on the structural transformation of individual factors; 3, and considered the macro level factors on the spreads effect, without considering other unobservable factors. But in this paper, the influence of micro level and the unobservable factors on the model is controlled by the change of bonds spreads.

The remainder of this paper is structured as follows. Section 2 builds Markov regime switching model based on GED according to asset pricing theory; Section 3 empirically studies the impact of macro economy, monetary policy, stock market and investor sentiment on bonds spreads in the context of structural transformation; Section 4 summarizes the results and gives a conclusion.

## Methods

### Spreads and macro-conditions

According to the theory of asset pricing [21], the expected yield of financial asset depends on the relationship between asset and market, represented by

$$E\left(R\right)=R_{f}-R_{f}\mathrm{cov}\left(m,R\right)=R_{f}+\left(\frac{\mathrm{cov}\left(R,m\right)}{\mathrm{var}\left(m\right)}\right)\left(-\frac{\mathrm{var}\left(m\right)}{E\left(m\right)}\right)=R_{f}+\beta \cdot \lambda_{m}$$ (1)

where *R*_*f*_ = 1/*E*(*m*) is the risk-free rate, *m* is a random discount factor related to aggregate consumption that reflects the macro risk, *β* = cov(*R*,*m*)/var(*m*) indicates the relationship between asset return and system risk, which reflects the risk of asset specificity, and *λ*_*m*_ = −var(*m*)/*E*(*m*) can be interpreted as the system risk price, determined by the risk aversion of market investors and the fluctuation of aggregate consumption, which is the result of macroeconomic condition. Therefore, the yields of corporate bonds and government bonds can be expressed as

$$E\left(R^{C}\right)=R_{f}+\beta^{C}\cdot \lambda_{m}\;and\;E\left(R^{T}\right)=R_{f}+\beta^{T}\cdot \lambda_{m}$$ (2)

Then the spreads in practical application is given by

$$spread=E\left(R^{C}\right)-E\left(R^{T}\right)=\left(\beta^{C}-\beta^{T}\right)\cdot \lambda_{m}=\tilde{\beta }\cdot \lambda_{m}$$ (3)

According to Eq (3), in the empirical study of bonds spreads theory, spreads can be regarded as the result of the joint action of market risk *λ*_*m*_, corporate bonds and government bonds. Depending on investors' risk attitude and social consumption volatility, market risk is affected by macroeconomic variables such as economic growth, monetary policy and investor sentiment [22–24]. This is consistent with the results of most studies that examined macro conditions and spreads [8, 25–28].

### Regime switching model

Many attempts have been made to confirm the impact of macro-level factors on the level and volatility of bonds spreads. In fact, in a certain time span, some factors may have structural changes in the impact on spreads due to cyclical or institutional changes, while the impact of other factors may be relatively stable. Therefore, this paper assumes the corresponding econometric model as

$$y_{t}=\alpha_{S_{t}}+\sum_{i=1}^{N_{nS}}\beta_{i}x_{i,t}^{nS}+\sum_{j=1}^{N_{S}}\phi_{j,S_{t}}x_{j,t}^{S}+\varepsilon_{S_{t}}$$ (4)

where $x_{i,t}^{nS}$ indicates the explanatory variables without structural changes, and $x_{j,t}^{S}$ are the explanatory variables with structural switching, that is to say the intercept and slope of the model are allowed to be alterable.

In some cases, the financial time series do not conform to the Gaussian distribution, but show the characteristics of skewness and kurtosis. The generalized error distribution (GED) proposed by JP Morgan in Risk Metrics can flexibly reflect this characteristic of time series, and its density function is given by

$$f\left(x|\mu ,\sigma ,r\right)=\frac{r\Gamma {\left(3/r\right)}^{1/2}}{2\sigma \Gamma {\left(1/r\right)}^{3/2}}\exp-{\left[\frac{\Gamma \left(3/r\right)}{\Gamma \left(1/r\right)}{\left(\frac{x-\mu }{\sigma }\right)}^{2}\right]}^{\frac{r}{2}}$$ (5)

If *r* = 2, then *GED*(*μ*,*σ*^2^,2) = *N*(*μ*,*σ*^2^). In this paper, GED is adopted to characterize the distribution properties of bonds spreads, i.e. $\varepsilon_{S_{t}}∼GED\left(0,\sigma_{S_{t}}^{2},r_{S_{t}}\right)$. In the model, it is assumed that shape parameter $r_{S_{t}}$ and variance $\sigma_{S_{t}}^{2}$ are both alterable. Let *S*_*t*_ = {1,2} be unobservable state variables, where *S*_*t*_ = 1 represents a high state (H_State), on behalf of a normal period, and *S*_*t*_ = 2 represents a low state (L_State), denoting an abnormal period with larger spreads volatility. It is assumed that the transitions between states follow a two-state Markov process, and the transitions matrix is given by

$$P=\left(\begin{array}{cc} p_{11} & p_{21}\\ p_{12} & p_{22} \end{array}\right)$$ (6)

where state transition probability is *p*_*ij*_ = *p*(*S*_*t*_ = *j*|*S*_*t*−1_ = *i*), *i*,*j* = 1,2, and *p*_*i*1_ + *p*_*i*2_ = 1.

Assuming that the state variables *S*_*t*_ at any time are unobservable, *y*_*t*_ can be observed and the probability of state *S*_*t*_ can be inferred through the evolution of *y*_*t*_, which is

$$\xi_{jt}=\mathrm{Pr}(S_{t}=j|\Omega_{t};\theta ),\;j=1,2.$$ (7)

where Ω_*t*_ = {*y*_*t*_,*y*_*t*−1_,…,*y*_2_,*y*_1_} represents the information set to time *t*, and *θ* is the parameter vector of the model. According to Hamilton’s (1994) filter, the state probabilities can be speculated using the information at *t* = 1,2,…,*T*. It can be assumed that the initial value of the iteration is *ξ*_1,0_ = *ξ*_2,0_ = 1/2, or the non-conditional probability of *S*_*t*_, namely, $\xi_{1,0}=\frac{1-p_{22}}{2-p_{11}-p_{22}}$, $\xi_{2,0}=\frac{1-p_{11}}{2-p_{11}-p_{22}}$.

The density function of *y*_*t*_ at different states is expressed as

$$\eta_{j\;t}=f\left(y_{t}|s_{t}=j,\Omega_{t-1};\theta \right)$$ (8)

where *j* = 1,2 represent the states. According to the Markov property, the conditional density of *t-th* observation is calculated by

$$f\left(y_{t}|\Omega_{t-1};\theta \right)=\sum_{j=1}^{2}\xi_{jt}\eta_{j\;t}=\sum_{i=1}^{2}\sum_{j=1}^{2}p_{ij}\xi_{i,t-1}\eta_{jt}$$ (9)

And then

$$\xi_{jt}=\frac{\sum_{i=1}^{2}p_{ij}\xi_{i,t-1}\eta_{jt}}{f\left(y_{t}|\Omega_{t-1};\theta \right)}=\frac{\sum_{i=1}^{2}p_{ij}\xi_{i,t-1}\eta_{jt}}{\sum_{i=1}^{2}\sum_{j=1}^{2}p_{ij}\xi_{i,t-1}\eta_{jt}}$$ (10)

Through the above iteration, the state probabilities from *t* = 0 to *t* = *T* are derived. Then, the log-likelihood function of the observed samples can be calculated by

$$ln\;f\left(y_{1},\;y_{2},\ldots ,\;y_{T}|y_{0};\theta \right)=\sum_{t=1}^{T}\ln f\left(y_{t}|\Omega_{t-1};\theta \right)$$ (11)

where parameters to be estimated are *θ* = *θ*(*p*_11_,*p*_22_,*σ*_1_,*σ*_2_,*r*_1_,*r*_2_,*β*_*i*_,*ϕ*_*j*_).

Using maximum likelihood estimation, we can obtain the estimated value of the parameters $\hat{\theta }$, as well as the time series of high (or low) state conditional probability *P*(*s*_*t*_ = 1|Ω_*t*_;*θ*). The expected duration of the two states is also obtained, i.e.d ${duration|}_{s_{t}=i}=\frac{1}{1-p_{ii}}$, *i* = 1,2.

In this way, the transformation characteristics of model structure can be estimated and the model structure in different states can be given, facilitating the analysis on the explanatory power of explanatory variables under different state conditions.

## Results

### Data

The main purpose of this paper is to investigate the comprehensive effect of market and macro conditions on the change of bonds spreads and the influence of different structures in the context of potential structural transformations. Considering the magnitude of the spreads variation, this paper takes 100 times (1% basis points) of the variation in the average bonds spreads as an explanatory variable (Δ*spread*_*t*_). By convention, bonds spreads are defined as the difference between the maturity yield of corporate bonds and the maturity yield of Treasury bonds with same maturity.

In order to elaborate the previous discussion, the influencing factors considered in this paper include macroeconomic performance, stock market, money supply and investor sentiment. Year-on-year GDP growth is adopted to indicate macro economy (*g*). The macroeconomic performance reflects the systemic aggregate risks of environment in which the business is located. Poor macroeconomic conditions on the one hand means that the enterprise will face greater uncertainty, the enterprise's future cash flow risk will increase, and the cash flow discount rate will increase too, along with the increasing liquidation costs. On the other hand, bad conditions will affect the consumption growth and increase the systematic risk of financial market. All of these will lead to the rise of bonds spreads. The weekly changes of Shanghai Composite Index (*Si*) is chosen to be the proxy of stock market. The stock market reflects investors' judgment of the future economic environment as well as the expectation for the economic operation. As the investment objects, stock market and bonds market are alternative, there is a transfer of risk and investment between the two markets. The volatility of the stock market has been demonstrated to inevitably affect the bonds market. Year-on-year currency growth is chosen as money supply indicator (*M*). The increase of money supply will help improve the business environment of enterprises, reduce the yield of bonds and affect the bonds spreads. At last, the index of investor confidence is adopted to represent investor sentiment (*Ci*).

Many factors affect bonds spreads, so this study uses the changes of the spreads in previous period to control the impact of unobserved factors. In order to control the impact of industry factors on bonds spreads, this study selects 336 manufacturing corporate bonds in China’s bonds market as the research object. Compared with other industries such as consumption, communications and services, manufacturing is more sensitive to macro factors. For controlling the impact of the bonds quality, the bonds are divided into four groups according to their ratings, namely 76 AAA bonds, 54 AA+ bonds, 168 AA bonds and 38 AA- bonds. Table 1 gives the statistical results of the average spreads of each group of bonds, showing that each group of data does not match Gaussian distribution. The JB test also rejects the normality assumption of spreads changes. Table 2 gives the statistics of macro data. The sample period of the study is 2013.5.10 to 2017.4.14, with weekly collection. For the difference in data release cycle, the paper adopts a linear interpolation method.The data come from Wind database.

**Table 1:** Summary statistics for average spreads of different credit grade.

	Mean	Std. Dev.	Min	Median	Max	Skewness	Kurtosis
AAA	0.0138303	0.003719	0.007074	0.0143121	0.031	0.2947494	3.766113
AA+	0.0201429	0.0044178	0.0137224	0.0194764	0.0305726	0.4821924	2.271951
AA	0.0268046	0.0050922	0.0203658	0.0203658	0.0418722	0.8830414	3.045388
AA-	0.046469	0.0069938	0.0289237	0.0725822	0.0725822	0.4260157	4.309557

**Table 2:** Summary statistics for macro-variables.

	Mean	Std. Dev.	Min	Median	Max	Skewness	Kurtosis
*g*_*t*_	0.0715049	0.0038513	0.067	0.07	0.078	0.4076646	1.778729
*Ci*_*t*_	0.0000294	0.0146057	-0.04575	-0.0106	0.0305	-0.700771	4.195348
*Si*_*t*_	0.0004946	0.014701	-0.074	0.0013	0.0482	0.3379099	10.76023
*M*_*t*_	0.1195343	0.0734491	0.012	0.0078	0.254	0.5636944	1.895297

### Empirical results

**Fig 1:**
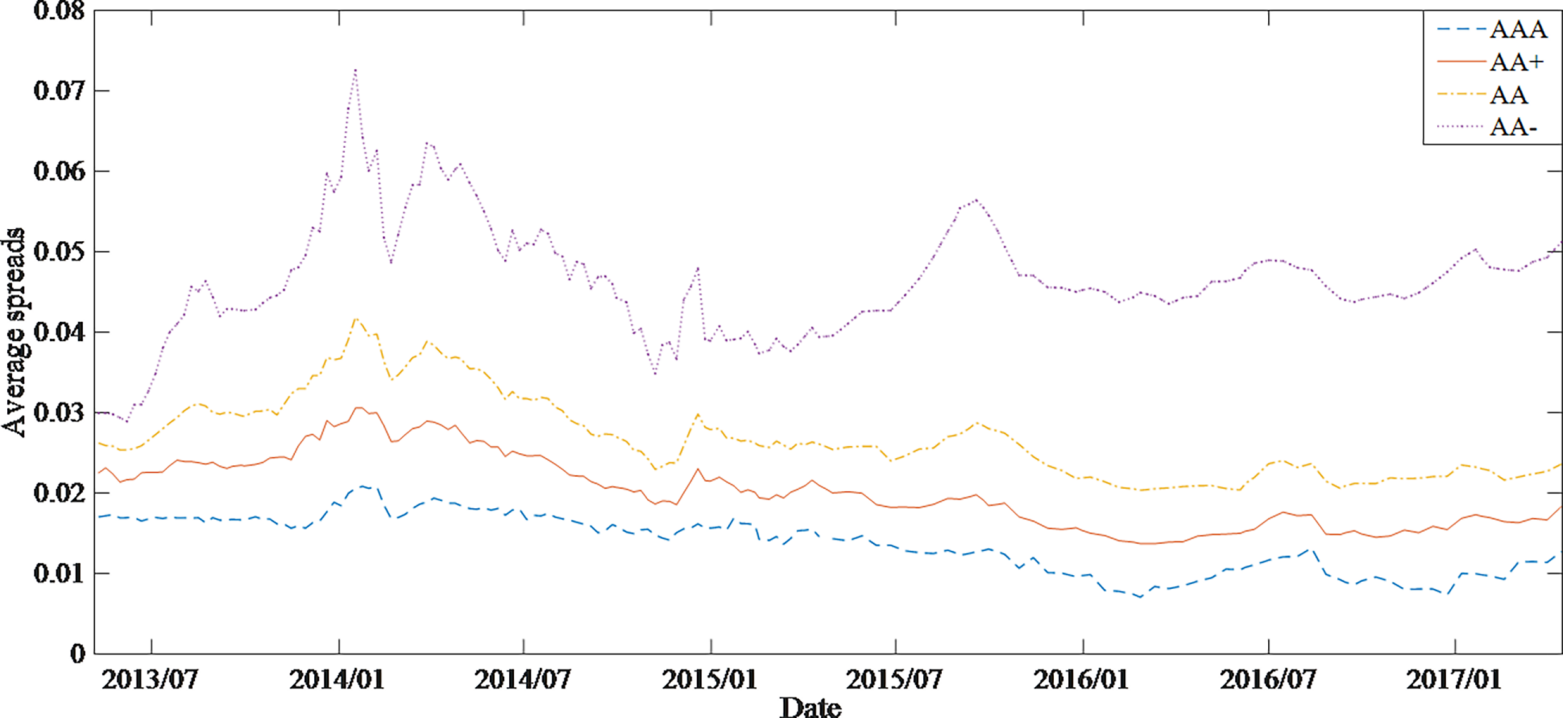
Average spreads of 4 group bonds from May 2013 to April 2017.

**Fig 2:**
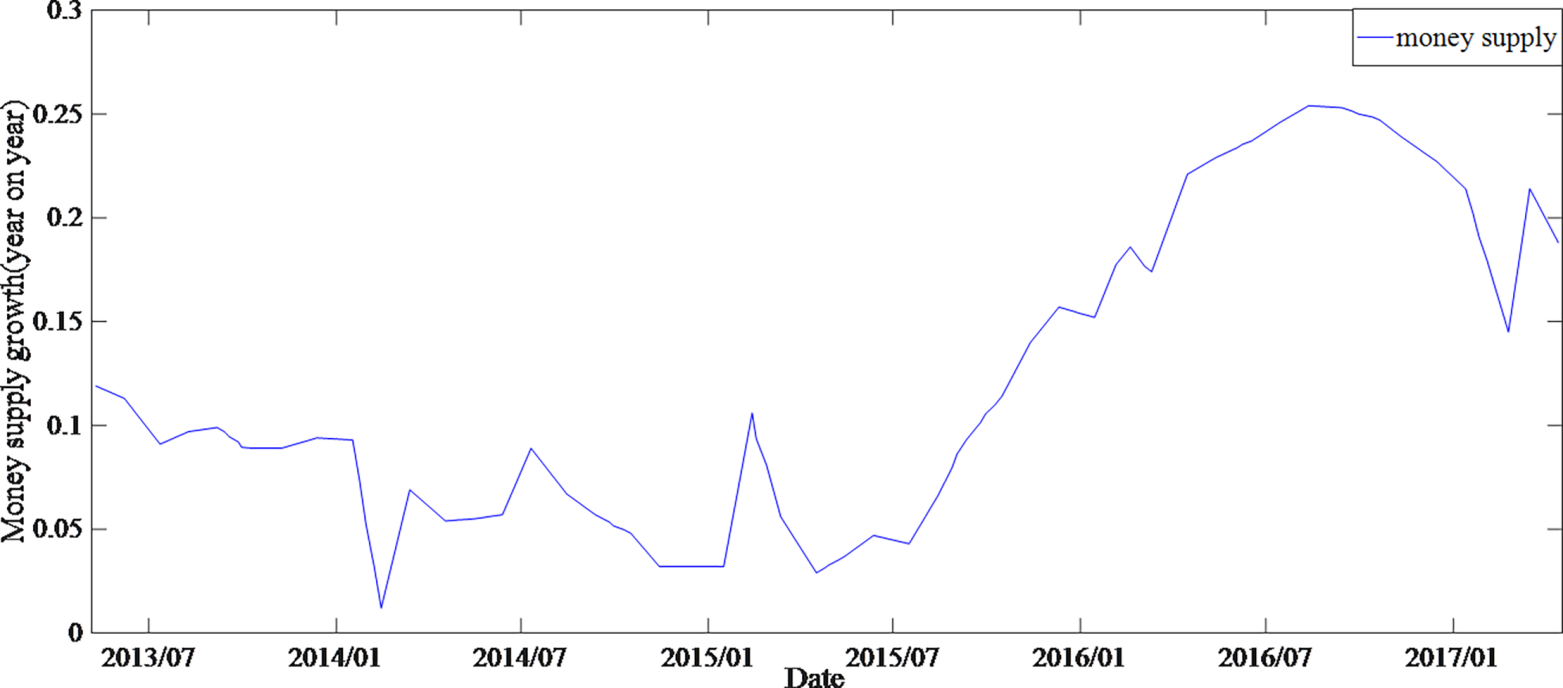
Money supply (M1) from May 2013 to April 2017, which reflects the China's monthly currency growth year on year.

**Fig 3:**
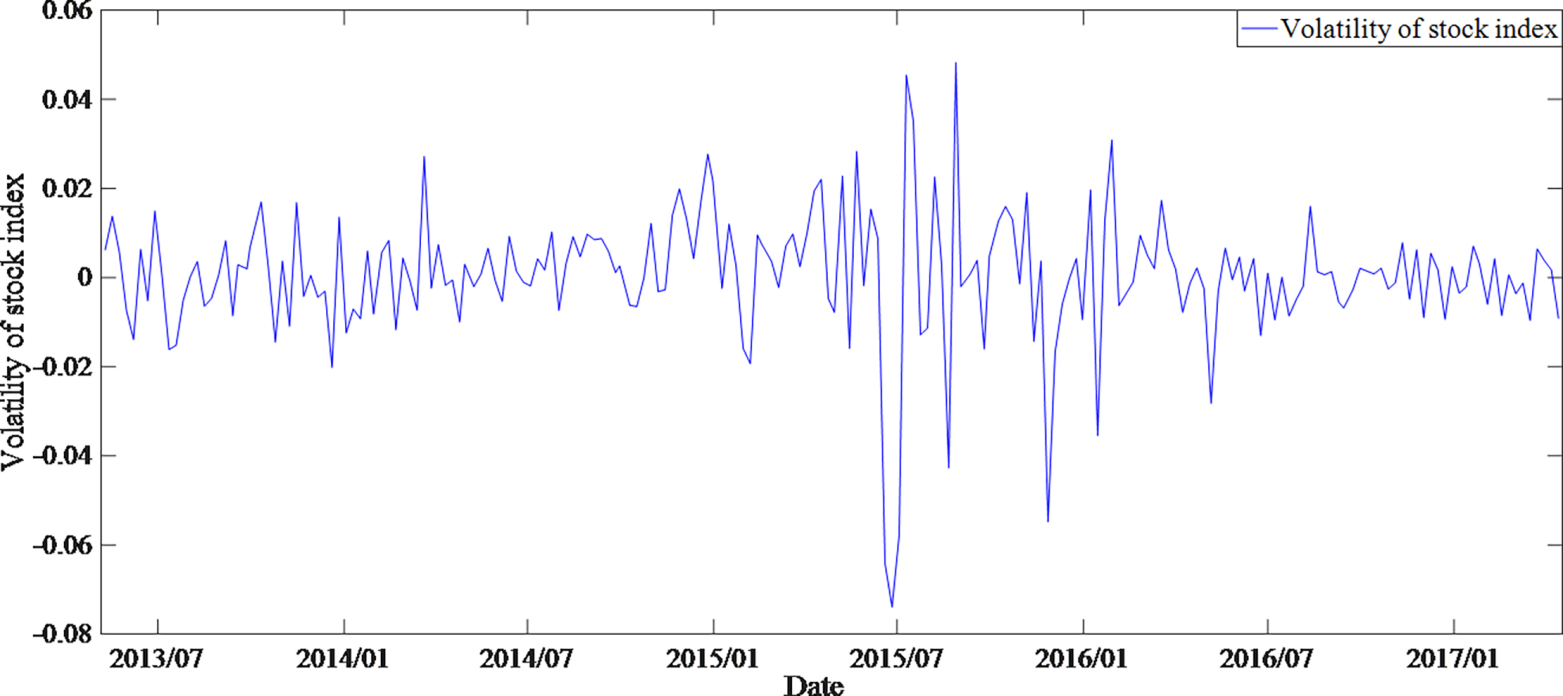
The volatility of Shanghai composite index.

By comparing the average spreads of different groups of bonds and the time series of relevant explanatory variables, it is found that higher-rated bonds not only have lower spreads but also have smaller spreads volatilities. In general, the variation trend of spreads is strongly correlated with the change of money supply and the volatility of stock market (Figs 1–3).

The increase of money supply and the fluctuation of the stock market will lead to the decrease of both the bonds yields and the spreads. Comparing the time series of bonds spreads and money supply, it is found that the money supply increased significantly after August 2015, but the decline in corporate bonds spreads during the same period was small. The average bonds spreads of grade AA- even had a certain rise, which was contrary to the financial theory (note: in the end of 2013, due to default aggregation, investor risk aversion increased, leading to a significant increase in bonds spreads.). It can be found from Fig 3, the stock market experienced a great fluctuation after December 2014. For hedging purposes, investors will shift the investment from stock market to bonds market, resulting in a higher demand for bonds, which in turn leads to higher bonds prices and lower spreads. However, Fig 1 shows a different situation. Therefore, it is assumed that there is a structural change in the interpretation of bonds spreads by money supply and stock market.

The cyclicity and volatility characteristics of macro economy are not obvious in a short period, and investors also have a reasonable expectation about the economy conditions. Therefore, it is assumed that there is no structural change in setting the macroeconomic indicators (*g*). The bonds market and investor sentiment usually have cyclical characteristics, so an alterable model structure with Δ*spread*_*t*_ and *Ci* is set in this paper. This paper focuses on studying the impact of macro-level factors on bonds spreads, but there are also periodic structural changes that may not be taken into account, so the intercept is also assumed to be changeable.

Relevant econometric model is set as follows

$$\Delta spread_{t}=\alpha_{S_{t}}+\beta_{1,S_{t}}\Delta spread_{t-1}+\beta_{2}g_{t}+\beta_{3,S_{t}}Ci_{t}+\beta_{4,S_{t}}Si_{t}+\beta_{5,S_{t}}M_{t}+\varepsilon_{S_{t}}$$ (12)

where $\varepsilon_{S_{t}}∼GED\left(0,\sigma_{S_{t}}^{2},r_{S_{t}}\right)$. The parameters to be estimated are

$$\theta =\theta \left(\alpha_{i},\beta_{1,i},\beta_{2},\beta_{3,i},\beta_{4,i},\beta_{5,i},\sigma_{i},r_{i},p_{11},p_{22}\right),i=1,2$$ (13)

In order to demonstrate the advantages of the model in capturing the effects of variables, a consistent structural model was set as a benchmark model for comparison, namely

$$\Delta spread_{t}=\alpha +\beta_{1}\Delta spread_{t-1}+\beta_{2}g_{t}+\beta_{3}Ci_{t}+\beta_{4}Si_{t}+\beta_{5}M_{t}+\varepsilon_{t}$$ (14)

Estimated results are shown in Table 3:

**Table 3:** Parameter estimation under fixed structure model.

	*α*	Δ*spread*_*t*−1_	*g*	*Si*	*Ci*	*M*	*σ*	*r*
AAA	0.0455 (0.00)	-0.0940 (0.00)	-0.5640 (0.00)	-0.1812 (0.08)	-0.0342 (0.26)	1.3787 (0.06)	0.000164 (0.19)	0.8842 (0.00)
AA+	-0.0160 (0.78)	0.0440 (0.08)	0.8096 (0.12)	-0.3567 (0.00)	-0.0826 (0.01)	0.6836 (0.29)	0.003342 (0.00)	0.6899 (0.00)
AA	-0.3194 (0.10)	0.2799 (0.00)	3.1085 (0.15)	-0.5192 (0.00)	0.1162 (0.20)	3.0767 (0.07)	0.004919 (0.01)	0.4159 (0.00)
AA-	-0.5868 (0.00)	0.1786 (0.00)	8.0792 (0.00)	-1.2044 (0.03)	0.2507 (0.00)	-1.845 (0.18)	0.027240 (0.01)	0.6784 (0.00)

(p-values are reported in parentheses)

Table 3 shows that the generalized error distribution hypothesis relative to the Gaussian distribution can better fit the data (regression analysis based on normal distribution is not presented here due to space limitations). However, under the fixed structure, many variables are not significant, which indicates fixed structure model cannot sufficiently explain the influence of macro-level factors on the spreads. At the same time, even if there is a significant explanatory power of variables, this ability is still an "average" effect within a certain period of time and cannot reflect more detailed information. This shortcoming becomes even more pronounced when the time series span is longer. To this end, the regime switching model based on generalized error distribution is adopted to solve this problem. The model is estimated according to previous research [29] and the results are shown in Table 4.

**Table 4:** Parameter estimating based on GED-MRS.

		*α*	Δ*spread*_*t*−1_	*g*	*Si*	*Ci*	*M*	*σ*	*r*
AAA	H_State	-0.0482 (0.00)	-0.1618 (0.00)	-0.0907 (0.00)	0.2955 (0.00)	-0.2376 (0.00)	7.1559 (0.00)	0.000173 (0.50)	0.8555 (0.03)
	L_State	0.0544 (0.00)	0.0745 (0.00)		-0.2264 (0.00)	-0.5030 (0.00)	-1.4981 (0.00)	0.000441 (0.42)	1.1761 (0.00)
AA+	H_State	-0.7907 (0.00)	-0.2590 (0.00)	7.5377 (0.00)	-1.4176	2.1282	7.4496	0.002088	0.0100
					(0.00)	(0.00)	(0.00)	(0.00)	(0.91)
	L_State	-0.7245 (0.00)	0.1021 (0.07)		-0.1378 (0.48)	1.3795 (0.23)	4.5522 (0.00)	0.002258 (0.17)	0.7642 (0.00)
AA	H_State	-0.5975 (0.00)	0.0802 (0.00)	5.4164 (0.00)	-0.9951 (0.00)	3.7152 (0.00)	2.5705 (0.00)	0.003357 (0.33)	0.8004 (0.00)
	L_State	-0.5190 (0.00)	0.2212 (0.00)		-0.1315 (0.00)	0.6917 (0.00)	6.8854 (0.00)	0.006844 (0.00)	0.0100 (0.94)
AA-	H_State	-0.1440 (0.00)	0.1146 (0.00)	3.3174 (0.00)	-5.5539 (0.00)	2.3011 (0.00)	-21.3834 (0.00)	0.002457 (0.00)	1.3866 (0.00)
	L_State	-0.2301 (0.00)	0.4176 (0.00)		-0.5854 (0.05)	-0.1120 (0.95)	2.6624 (0.05)	0.023517 (0.01)	0.3671 (0.00)

Note: H_State denotes the regimes with low volatility, and L_State represents the regimes with high volatility. (p-values are reported in parentheses)

The empirical results show that all groups of bonds experience significant structural transformations, especially the low-rated AA- bonds, which have very different factor loadings between two states. The alternative model based on Gaussian distribution is also considered for corresponding empirical analysis and it is found that the regime switching model based on GED has better performance. Among the explanatory variables that have structural changes, there are significant differences in factor loading between high state (H_State) and low state (L_State), and the coefficients of some explanatory variables may even appear opposite signs under different states. For example, for the AAA bonds in low state, the bonds spreads will be negatively correlated with the volatility of the stock market, and in high state, the relationship becomes a positive one. The same holds true for the coefficients of money supply (*M*_*t*_) on AAA and AA- bonds. This suggests that using structural switching models can capture the behavior differences masked by the "averaging" of invariant structural models, and also reveal more clearly the effect of explanatory variables on dependent variables.

The spreads of high-grade bonds (AAA and AA+) are found to show significant recursive characteristics (negative coefficients of Δ*spread*_*t*−1_) at normal state, while other cases show a sustainability feature (positive coefficients of Δ*spread*_*t*−1_), and the latter property continues to significantly enhance with the decline of bonds quality. This reflects the fact that in China's bonds market, only the price of high-grade bonds shows a rational fluctuation under normal circumstances, and investors use it as an investment target. The price dynamics of low-rated bonds are more of a reflection of investors' speculative psychology, and the speculative mentality become even stronger as bonds ratings fall and market conditions deteriorate.

The impact of macroeconomic indicator (*g*) on bonds spreads displays an interesting phenomenon. On the one hand, macroeconomic indicators have little impact on the prices of high-grade bonds, suggesting that the fundamentals of high quality bonds are less volatile with the macro economy. At the same time, it also reflects the market recognition for the prices of high-grade bonds, and there is a stable demand for good bonds. On the other hand, for low-rated bonds, the role of macroeconomic conditions decreases with the downgrade of the bonds rating. More importantly, the coefficient sign of explanatory variables is significantly positive. These fully reflect that the size of China's bonds market is relatively smaller than other markets such as the stock market, and the bonds market is more vulnerable to the impact of other capital markets. When the economy goes up, other financial markets have higher yields, and their boom will cause the transfer of investment, resulting in a relative decrease in bonds market investment, and a widening of the spreads.

The stock market is the most important component of China's financial system and it has obvious influence on corporate bonds market. It can be seen from Table 4 that the impact of the stock market on AAA bonds is significantly different from that of other groups. For AAA bonds, under the high-state structure, the boom in the stock market will result in an increase in spreads, reflecting the investment substitution between the two markets. In other cases, however, the coefficients are negative, indicating that the stock market is more indicative of the overall business environment. In high-state (i.e. normal state) structure, especially for low-rated bonds, spreads are more sensitive to the stock market and stock market is more indicative of economic conditions. Combing with the macroeconomic indicators, it can be deduced that the boom of the stock market generally reflects the boom of macro economy, manifests the improvement of the fundamentals of enterprises, and conducive to reduce the risk price of bonds. However, when the economy is booming, the relatively-low yield of the bonds market can cause outflow of funds, resulting in underinvestment in the bonds market. This will lead to a decline in the bonds price and a rise in the yield to maturity, which ultimately reflects the upward floating of the spreads. The strength of substitution and indication is different for different grades of bonds in different states.

For different grades of bonds, investor sentiment indicator *Ci*_*t*_ shows a great difference. There are significant structural differences in the impact of investor sentiment on AAA and AA bonds, but not for AA+ and AA- bonds. The effect of sentiment indicators varies widely across bonds groups and increases as the bonds rating declines, reflecting the different preferences of investors for different grades of bonds, which are also significantly affected by market conditions. It can be seen from Table 4 that investors are more likely to invest in medium-risk bonds such as AA+ and AA.

Money supply has a significant impact on the spreads of each group of samples. After all, the financial markets are more directly tied to money supply. These effects are also characterized by significant structural changes, and their structures appear to be reversed for high-rated (AAA, AA+) and low-rated (AA, AA-) bonds. Under normal circumstances, money supply has a relatively greater impact on high-quality bonds; while under low conditions, money supply has a greater impact on low-rated bonds. This shows that the risk preference of investors to different grades of bonds varies with the market conditions, which further reflects the phenomenon of investment diversion among different classes of bonds.

**Fig 4:**
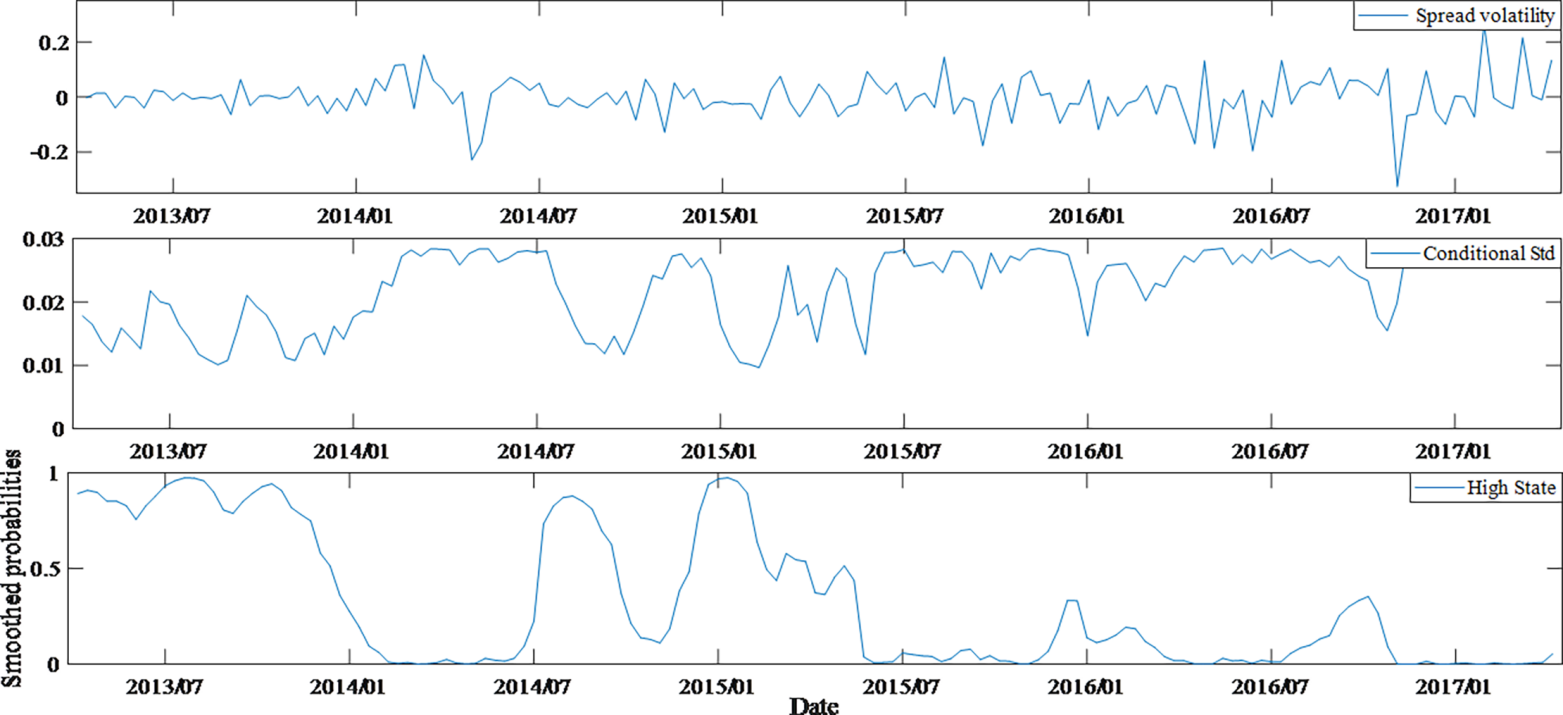
Dynamic feature of average spreads, conditional variances and high state probabilities for AAA bonds.

**Fig 5:**
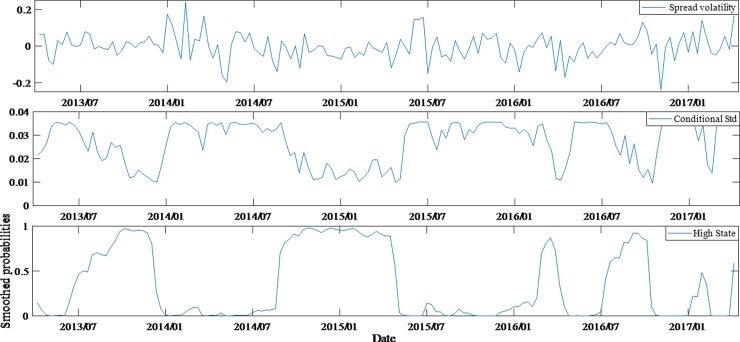
Dynamic feature of average spreads, conditional variances and high state probabilities for AA+ bonds.

**Fig 6:**
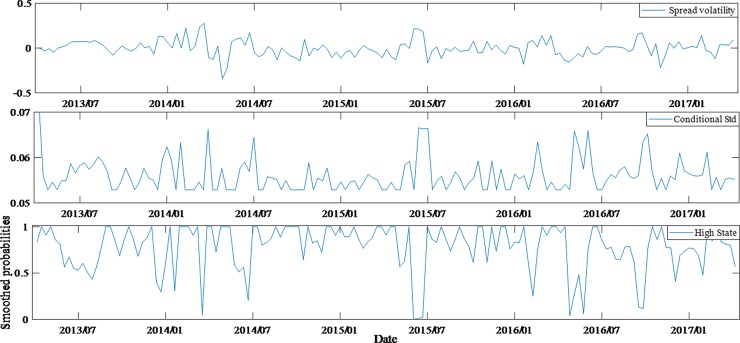
Dynamic feature of average spreads, conditional variances and high state probabilities for AA bonds.

**Fig 7:**
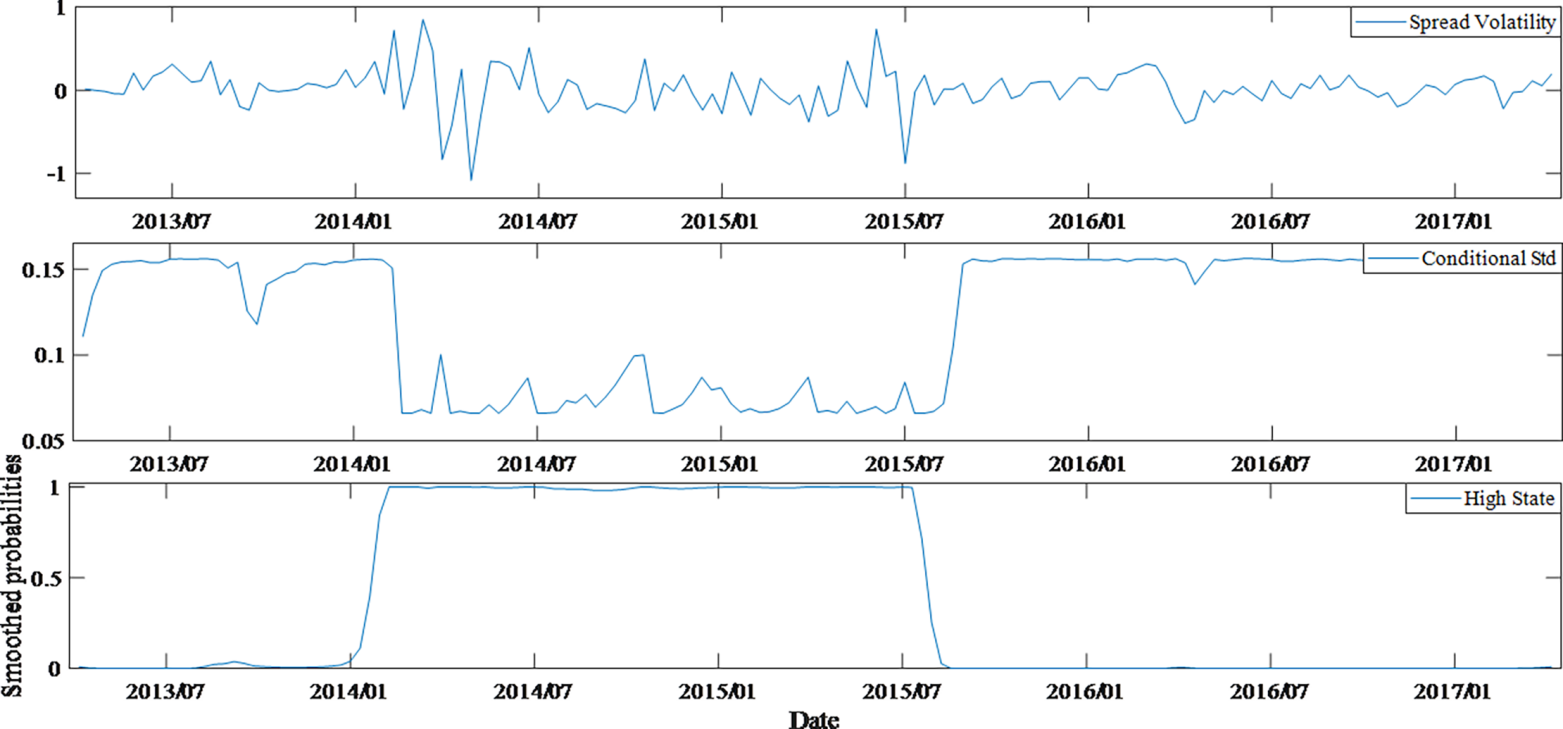
Dynamic feature of average spreads, conditional variances and high state probabilities for AA- bonds.

According to the comparison of estimated results, it can be seen that regime switching model performs better than invariant structural model, as the former can also statically reflect the changes in the impact of variables on spreads under different states. Furthermore, by comparing the volatilities of spreads, conditional variances and the probability time series of the state (high state) for different ratings bonds (Figs 4–7), we can also have knowledge about the dynamic characteristics of spreads structure.

Through the above comparison, it is found that the lower the rating of bonds, the more obvious the structural transformation features and the stronger the stability of the structure. In fact, through the estimation of Markov process, the state transition matrix of AAA bonds and AA- bonds are acquired, being $\left(\begin{array}{ll} 0.85 & 0.06\\ 0.15 & 0.94 \end{array}\right)$ and $\left(\begin{array}{ll} 0.98 & 0.01\\ 0.02 & 0.99 \end{array}\right)$ respectively.

Therefore, the probabilities of the AAA bonds being stable in high and low states are 0.85 (*p*_11_) and 0.94 (*p*_22_) respectively, the corresponding probabilities of AA- bonds are 0.98 and 0.99. The difference can be found from Figs 4 and 7. According to the state duration formula *T*_*i*_ = 1/(1−*p*_*ii*_), *i* = 1,2, it can be concluded that the expected duration of AAA bonds is 16.55 weeks (low state) and 6.82 weeks (high state), while the AA- bonds is 83.42 weeks and 50.02 weeks, respectively.

The figures also reflect the non-synchronization of the state transitions in each group of bonds. Before January 2014, AA-rated bonds had a distinctly different state from AAA and AA+ bonds. In particular, from November 2013 to January 2014, the AAA and AA+ bonds were in a high-state structure period, while the AA- bonds were in low-state structure period. This shows that a shift is found in the investment both between different markets and between different grades of bonds. However, since January 2014, the bonds market risk events had been frequent and investors' worries about credit risk began to rise significantly. That would lead to a surge in the corporate bonds spreads in the same period. In fact, this was the third time in history that the spreads rose sharply, with the first two being the 2008 financial crisis and 2011 Quasi-municipalBonds crisis. It is obvious that huge external shocks masked the structural changes. The state of AAA, AA+ and AA- bonds was basically in sync after January 2015. During the entire sample period, AA bonds had a more dispersed state period. In fact, their expected state durations were also the most average of four groups, which were 10.87 weeks (high state) and 11.59 weeks (low state) respectively. These reflect the investor's attitude towards different ratings of bonds, and also objectively reflect the investment transfer in the bonds market.

By comparing the figures, it is found that although the monetary policies and the development of the stock market after July 2015 were conducive to the decrease of bonds spreads, the four groups of bonds samples were basically in the low state, which largely offset the favorable news, so bonds spreads did not show a significant reduction. Especially, AA- bonds entered a low-state period after July 2015 and their spreads had higher continuity under low state. Therefore, it can be observed that the spreads of AA- bonds did not decrease after July 2015.

The economic cycle theory generally assumes that the economic environment has high and low states. Based on this, this paper also assumes that the model structure switches between two states. In order to verify the rationality of the two-state hypothesis, the paper also empirically analyzes the case of three states and four states. The results show that the three-state or the four-state assumption only increases the number of the model’s parameters, up to 28 and 41 respectively, but it does not really improve the model.

## Conclusions

Faced with the fat-tail financial data and cyclical characteristics of financial market, as well as incidental external shocks, the fixed structure model based on the Gaussian distribution cannot well depict the impact of macroeconomic conditions on bonds spreads, and cannot accurately capture the dynamic changes of factor loading.

Using the Markov regime switching model based on generalized error distribution, this paper analyzes the comprehensive impact of economic development, monetary policy, stock market and investor sentiment on the spreads of different grades of bonds in Chinese listed companies. The results show that in Chinese corporate bonds market, macro conditions have significant structural transformation characteristics to the impact of the spreads, and the characteristics are strengthened as the bonds rating declines, and meanwhile, the structural differences increase as the bonds rating decreases. The results show that the states of different ratings bonds are not synchronized, and even in opposite directions of successive months, which indicate the existence of investment transfer between different grades of bonds. The study also indicates that higher-grade bonds exhibit a value reversion at high state, but in other cases, spreads show significant persistence, which reflects investors’ attitude toward high-and low-grade bonds. Overall, China's corporate bonds spreads are significantly influenced by macro conditions and the clustering of default events would lead to a surge of spreads, investors’ preferences for different ratings are significantly different as well. All these will help further understand the operating rules of bonds market, and formulate investment strategies and reasonable supervision.

## Supporting information

S1 Data This is the data set for the research. (RAR)
